# A translational preclinical model of interstitial pulmonary fibrosis and pulmonary hypertension: mechanistic pathways driving disease pathophysiology

**DOI:** 10.14814/phy2.12133

**Published:** 2014-09-11

**Authors:** Elizabeth R. Jarman, Valerie S. Khambata, Li Yun Ye, Kenneth Cheung, Matthew Thomas, Nicholas Duggan, Gabor Jarai

**Affiliations:** 1 Respiratory Disease Area, Novartis Institutes for BioMedical Research, Horsham, West Sussex, UK

**Keywords:** Idiopathic pulmonary fibrosis, preclinical disease model, pulmonary hypertension, transforming growth factor‐*β*

## Abstract

Idiopathic pulmonary fibrosis (IPF) is a chronic progressive interstitial lung disease, in which a decline in patient prognosis is frequently associated with the onset of pulmonary hypertension (PH). Animal models exhibiting principle pathophysiological features of IPF and PH could provide greater insight into mechanistic pathways underlying disease progression and a means for evaluating novel therapeutic approaches for intervention. Here, we describe an in vivo disease model, in which animals develop progressive interstitial pulmonary fibrosis and associated PH, as defined by the presence of fibrotic foci adjacent to areas of alveolar injury and remodeling of the pulmonary vasculature. Associated changes in physiological parameters included a decline in lung function and increase in mean pulmonary arterial pressure (mPAP) >25 mmHg. The early fibrotic pathology is associated with a profibrogenic microenvironment, elevated levels of the matrix metalloproteases, MMP‐2, MMP‐7, and MMP‐12, TIMP‐1, the chemoattractant and mitogen, PDGF‐*β*, and the chemokines CCL2 and CXCL12, that are associated with the recruitment of macrophages, mast cells, and fibrocytes. Principle mechanistic pathways associated with disease pathogenesis are upregulated in the lungs and pulmonary arteries, with sustained increases in gene transcripts for the profibrotic mediator TGF‐*β*1 and components of the TGF‐*β* signaling pathway; PAI‐1, Nox‐4, and HIF‐1*α*. Therapeutic treatment with the ALK‐5/TGF‐*β *RI inhibitor SB‐525334 reversed established pulmonary fibrosis and associated vascular remodeling, leading to normalization in clinically translatable physiological parameters including lung function and hemodynamic measurements of mPAP. These studies highlight the application of this model in validating potential approaches for targeting common mechanistic pathways driving disease pathogenesis.

## Abstract (precis)

Idiopathic pulmonary fibrosis is a chronic progressive interstitial lung disease, in which a decline in patient prognosis is frequently associated with the onset of pulmonary hypertension (PH). Animal models exhibiting principle pathophysiological features of disease could provide greater insight into mechanistic pathways underlying disease progression and a means for evaluating novel therapeutic approaches for intervention. Here, we describe an in vivo disease model, in which animals develop progressive interstitial pulmonary fibrosis and hypertension and demonstrate upregulation in mechanistic pathways associated with disease pathogenesis, both in the lungs and pulmonary arteries.

## Introduction

Idiopathic pulmonary fibrosis (IPF) is a chronic progressive interstitial lung disease (ILD) of poor prognosis, for which there is no effective therapy available. There is a high incidence of pulmonary hypertension (PH) in IPF (Proceedings of the 4th World Symposium 2009) with a prevalence of between 28% and 46% and as much as 85% within preselected patient cohorts (Nathan et al. 2008). In both disease indications, diagnostic symptoms include dyspnea, fatigue, and exercise limitations. Consequently, PH may remain undiagnosed in patients with interstitial lung disease until a late stage in disease progression, usually marked by right heart failure and increased mortality. The underlying etiology in IPF is generally unknown, leading to the hypothesis that patients presenting with PH may represent a subgroup exhibiting a distinct clinical phenotype. Given the high incidence of PH in patients with IPF and the detrimental impact on disease progression, there is a need to identify common underlying molecular pathways involved in disease pathogenesis, potential biomarkers associated with disease progression and effective therapeutic approaches for intervention. Animal models that reflect key pathophysiological features of disease and demonstrate upregulation of principle molecular pathways provide a means of addressing these objectives.

The histopathology of IPF is characterized by usual interstitial pneumonia (UIP), as identified by heterogeneous interstitial fibrosis interspersed with fibrotic foci (American Thoracic Society, European Respiratory Society 2002). These are areas of active fibrosis, characterized by intense matrix deposition and the presence of contractile myofibroblasts, located adjacent to sites of alveolar epithelial cell injury – known as honeycomb cysts and lined with hyperplastic alveolar epithelial cells (Selman et al. 2011). PH is a pathophysiological parameter that occurs in a variety of clinical indications and is consequently associated with a range of histological abnormalities. The characteristic pathological features of PH in interstitial lung diseases, such as IPF, include remodeling of the small to medium distal pulmonary arterioles in the lung, with medial hypertrophy and intimal obstructive proliferation, resulting in increased pulmonary vascular resistance and ultimately right heart failure (Rabinovitch 2007). The clinical diagnosis of PH is defined by an increase in mean pulmonary arterial pressure (PAP) ≥25 mmHg at rest, as assessed by right heart catheterization (Galiè et al. 2009a).

Within the lung, gaseous exchange is facilitated by the close proximity of alveolar epithelial cells and endothelial cells, with fusion of their underlying basement membranes to form the alveolar capillary membrane. IPF is thought to arise due to successive injuries to this alveolar capillary membrane, resulting in loss of basement membrane integrity and an inability to reestablish normal lung architecture, thereby promoting fibrogenesis. In the absence of normal tissue regeneration, injured alveolar epithelial cells (AEC) undergo hyperplasia and apoptosis. Profibrotic mediators, such as TGF‐*β*, released by injured AEC, induce mesenchymal cell activation and deposition of ECM components within the alveolar space (Strieter 2005). Similarly, injury to microvascular endothelial cells leads to apoptosis, increased vascular permeability, an influx of plasma proteins, and the release of vasoconstrictors, pro‐inflammatory mediators, smooth muscle cell growth factors, and profibrogenic mediators (Morrell et al. 2009). Chronic injury to the alveolar capillary membrane and the release of mediators that promote the onset of pulmonary fibrosis and vascular remodeling may account for the development of PH in association with interstitial lung disease.

In response to the chronic hypoxic conditions that arise as a result of impaired gaseous exchange in the injured lung, adventitial fibroblasts surrounding pulmonary arteries have been shown to undergoing activation, proliferation, and differentiation to myofibroblasts. This is associated with increased matrix deposition around the vessel wall and the release of mediators that promote vascular remodeling. Within the vessel wall itself, microvascular endothelial cells and vascular smooth muscle cells (VSMC) undergo hyperplasia and hypertrophy (Stenmark et al. 2013).

The pathophysiology of IPF is thought to arise due to dysregulated tissue repair following injury, in the context of a profibrotic and potentially hypoxic microenvironment, characterized by an antioxidant–oxidant imbalance and elevated levels of reactive oxidative stress (ROS); conditions that predispose toward vascular remodeling and PH (Kinnula and Myllarniemi 2008). Impaired intraalveolar fibrinolysis provides a matrix for mesenchymal cell recruitment and expansion within the alveoli, as well as neovascularization. Elevated levels of growth factors and transcription factors, such as transforming growth factor (TGF)‐*β*1, platelet derived growth factor (PDGF), connective tissue growth factor (CTGF), and hypoxia‐inducible transcription factor (HIF)‐1*α* (Selman et al. 2011), promote mesenchymal cell expansion and matrix deposition, whereas the chemokines (C‐C motif) ligand (CCL)‐2/monocyte chemotactic protein (MCP‐1) and CXCL12/stromal cell‐derived factor‐1*α* (SDF‐1*α*) promote the recruitment of bone‐marrow‐derived inflammatory cells as well as endothelial and mesenchymal progenitor cells, thereby further promoting inflammation, fibrosis, vascular remodeling, and angiogenesis (Andersson‐Sjoland et al. 2008; Price et al. 2012; Yeager et al. 2012).

Numerous rodent models of pulmonary fibrosis (Degryse and Lawson 2011) or pulmonary (arterial) hypertension (Stenmark et al. 2009) have been established. However, there remains a lack of well‐characterized models in which the molecular pathways underlying the pathophysiology of both diseases are defined.

Studies have demonstrated that localized expression of high levels of the profibrotic mediator TGF‐*β*1 within the lungs of rodents results in the induction of pulmonary fibrosis and hypertension (Farkas et al. 2009). Here, we describe a rodent model, in which animals develop key pathophysiological features of both IPF and PH, following intratracheal challenge with the chemotherapeutic agent bleomycin. We demonstrate upregulation of principle components of pathways known to play a role in disease pathogenesis and show that the decline in lung function and increase in right ventricular pressure associated with established disease pathology can be attenuated and reversed by targeting these pathways. These data indicate that this model is appropriate for evaluating therapeutic approaches for patients with PH secondary to interstitial lung diseases such as IPF.

## Materials and Methods

### Animal model of bleomycin induced lung injury, fibrosis, and pulmonary hypertension

Animal care and experimental procedures were performed in accordance with Novartis and UK Home office animal ethics regulations. Groups of male Sprague‐Dawley rats, Charles River, UK (*n *= 8) were challenged under anesthesia by intratracheal instillation of bleomycin (3 mg/kg, Bleo‐Kyowa; Nippon Kayaku Co., Ltd, Tokyo, Japan) in 0.2 mL sterile isotonic saline using a PENN Century Microsprayer^™^ (EMMS, Hants, UK). Animals were dosed from day 7 onwards following bleomycin challenge, with the low molecular weight compounds; Imatinib mesylate/Gleevec (50 mg/kg/day) or the selective activin receptor–like kinase (ALK)‐5 inhibitor SB‐525334 (10 mg/kg/day) in vehicle (sterile deionized H_2_O or 0.5% Methylcellulose/0.5% Tween 80, respectively) administered by oral gavage. Control groups received vehicle as placebo (Grygielko et al. 2005; Manley et al. 2010).

### Lung function measurements

Measurements of lung function were obtained by forced maneuvers using the flexiVent^®^ (SIREQ Sientific Respiratory Equipment Inc., Montreal, QC, Canada). Animals were anesthetized with medetomidine (1 mg/mL) and ketamine (100 mg/mL), tracheotomized, and mechanically ventilated. Respiratory mechanics were determined by application of predefined pressure/volume perturbations to the airways. Dynamic readouts, including total lung capacity, resistance, and compliance were obtained by the linear single‐compartment model using multiple linear regression. Measurements of respiratory system input impedance; tissue damping and tissue elastance were obtained using multiple low‐frequency forced oscillations. All measurements were carried out until three acceptable readings (coefficient of determination >0.95) were recorded for each animal, and the average calculated.

### Hemodynamic measurements

Right ventricular systolic pressure was measured in anesthetized animals (ketamine and xylazine) by right heart catheterization through the right jugular vein (MPVS‐300 Systems; Millar, Houston, TX).

### Echocardiographic assessment

Transthoracic echocardiographic assessment was performed by ultrasound on sevoflurane‐anesthetized animals using the Vivid7 system and analyzed using the Echo PAC dimension software (GE Healthcare, Buckinghamshire, UK). To visualize the pulmonary artery (PA) outflow tract, a pediatric probe was placed in a parasternal lung axis position. Pulse flow Doppler imaging was used to observe the dynamics of blood flow through the PA valve. Changes in mid‐systolic notch (degree of indent through deceleration flow and measure of tricuspid regurgitation; score from 0 to 3) were determined for each animal. Motion mode analysis was used to measure right ventricular wall thickness in systole.

### Histology and Immunohistochemical staining of lung tissue

The left lobe of each lung was inflated with 10% formalin, fixed and embedded in paraffin. Parallel sections of tissue (3 *μ*m) were stained with Picro‐Sirius red (PSR) for collagen deposition or anti‐*α*‐smooth muscle actin (*α*‐SMA)‐specific mouse IgG2a monoclonal antibody (clone 1A4 1:4000; Sigma, Gillingham, UK). Macrophage infiltration was determined by staining with an antibody specific for CD68 (ab31630; Abcam, Cambridge, UK) and mast cells detected by staining with toluidine blue. To determine the extent of vascular remodeling, sections were costained for expression of *α*‐SMA and the epithelial cell marker von Willebrand factor (vWF). All sections were counterstained with hematoxylin, using the DAB Map detection system and Ventana Discovery XT Immunostainer (Ventana Medical Systems, Inc. Tuscon, AZ) and scanned (Aperio Scanscope T2; Aperio, Oxford, UK). The extent of the fibrotic pathology was assessed using the Ashcroft scoring system, with slides scored in a blinded fashion over an average 64 fields of vision. The degree of muscularization of pulmonary arteries within the lung (<50 *μ*m in diameter) was determined using image analysis software (image pro; Leica Microsystems, London, UK) and presented as percent *α*‐SMA staining of vWF‐positive vessels. The extent of vascular remodeling was assessed as degree of circumferential *α*‐SMA staining and categorized as nonmuscularized, partially or fully muscularized. Mean percent values are presented for an average of 64 fields of vision, covering the entire section of lung, with slides scored in a blinded fashion.

### Gene expression analysis

RNA was extracted from lung tissue or pulmonary arteries and TaqMan PCR performed, as described elsewhere (Humbert et al. 2004). Levels of gene expression were determined using the standard curve method, with samples run in triplicate. Primer and probe sequences are listed in Table 1.

**Table 1.** Reagents

Taqman^®^ Gene Expression Assay	Assay ID
Rat *β*‐2‐microglobulin	Rn00560865_m1
Rat HIF‐1*α*	Rn00577560_m1
Rat pro‐TGF‐*β*	Rn00572010_m1
Rat PAI‐1	Rn01481341_m1
Rat Fibronectin	Rn00569575_m1
Rat Collagen 3*α*1	Rn01437681_m1
Rat Collagen 1*α*1	Rn01463848_m1
Rat PDGF‐*α*	Rn00709363_m1
Rat PDGF‐*β*	Rn01502596_m1
Rat NOX‐4	Rn00585380_m1
Rat VEGFa	Rn01511601‐m1
Rat CTGF	Rn01537279_g1
Rat tph‐1	Rn01476869_m1
Rat IL‐6	Rn01410330_m1
Rat CCL‐2	Rn00580555_m1
Rat CXCL12	Rn00573260_m1
Rat MMP‐2	Rn01538170_m1
Rat MMP‐7	Rn00689241_m1
Rat MMP‐12	Rn00588640_m1

Primer and Probe sets used for TaqMan Gene expression assays were purchased from Applied Biosystems (Carlsbad, CA).

### Analysis of levels of profibrotic mediators

Cytokine levels in bronchoalveolar lavage (BAL) fluid were determined using commercially available assays, according to manufacturer's instructions; TGF‐*β*1 (Quantikine ELISA, MB100B; R&D systems, Abington, UK), rat TIMP‐1 (Duo Set ELISA, DY580; R&D systems), rat PAI‐1 (Total Antigen assay, RPAIKT‐TOT; Innovative Research, Peary Court Novi, MI). Values obtained were adjusted for total protein content using the Micro‐BCA Protein Assay Kit (Pierce, Thermo Scientific, Cramlington, UK).

### Hydroxyproline assay

Collagen deposition and accumulation in lung tissue, over time, was determined by measuring levels of the amino acid hydroxyproline. Tissue was hydrolyzed in 6 mol/L HCl at 120°C for 8–16 h, allowed to cool, and neutralized by the addition of phenolphthalein and 10 mol/L NaOH, before diluting in dH_2_0 and filtering through a 45 *μ*m filter (0.13 mm diameter). For the assay; 5‐*μ*L sample or hydroxyproline standard (5–500 *μ*g/mL) was added to 96‐well microtiter ELISA plates in triplicate, citrate acetate buffer (5 *μ*L) added to each well, followed by Chloramine‐T solution (100 *μ*L) and plates incubated at room temperature for 20 min. Ehrlich's reagent (100 *μ*L) was added and plates incubated at 65°C for a further 20–30 min, before obtaining colorimetric readings at 550 nm using a NanoDrop 8000 Spectrophotometer (ThermoScientific, Waltham, MA). Values were adjusted for protein content.

### Fibrocyte analysis

Flow cytometric analysis (FACS) was performed on single‐cell suspensions obtained from pooled blood samples (*n *= 4) using EDTA as anticoagulant. Nonspecific binding was inhibited using Fc blocking antibody (Rat Fc block/anti‐rat CD32 (BD Biosciences, Oxford Science Park, UK) and cells incubated with directly conjugated primary antibodies; Ms anti‐rat CD45 Alexa Fluor 647 (BioLegend, Cambridge Biosciences, UK), or isotype control; MsIgG1 Alexa Fluor 647 (BioLegend), washed, permeabilized using CytoFix/Perm kit (BD Biosciences, Oxford Science Park, UK), and then incubated with a biotinylated collagen I‐specific primary antibody Biotin ab6577 (Abcam) or isotype control rabbit polyclonal IgG (Southern Biotech, Cambridge Biosciences, UK) followed by Streptavidin‐PE (BD Biosciences). Samples were analyzed in duplicate. Data are presented as total number of cells after adjusting for cell count using trypan blue exclusion.

### Statistical analysis

Unless indicated, statistical analysis was performed using one‐way ANOVA with Bonferroni multiple comparison (GraphPad Prism 5 Software, La Jolla, CA).

## Results

### Bleomycin induces progressive interstitial pulmonary fibrosis in rats

**Figure 1.**
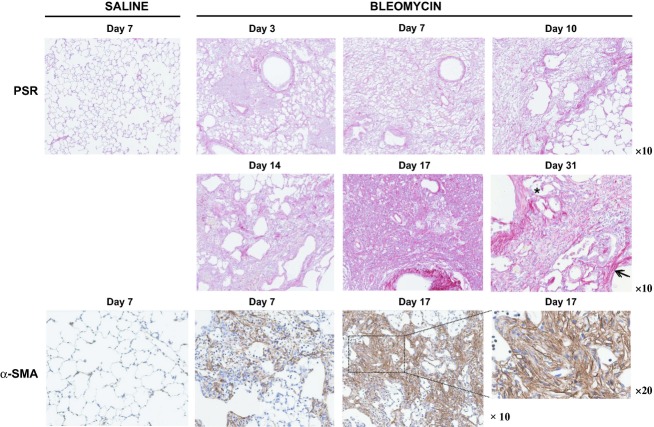
Morphometric analysis demonstrating marked collagen fiber deposition and *α*‐SMA‐expressing myofibroblasts within the lungs of bleomycin‐challenged rats. Sections of lung tissue isolated from groups of saline‐ or bleomycin‐challenged rats (*n *= 8) at various time points were stained with either Picro‐Sirius red (PSR) for collagen or with a *α*‐SMA‐specific antibody to detect the presence of contractile myofibroblasts within fibrotic foci. Sections were counterstained blue with hematoxylin. Representative samples are shown at ×10 and ×20 magnification.

Bleomycin is a chemotherapeutic agent that has the potential to cause interstitial lung disease in humans. The response generated in rodents is both species and strain dependent. Here, we demonstrate that a single intratracheal instillation of bleomycin into the lungs of Sprague Dawley rats leads to progressive interstitial pulmonary fibrosis. The fibrotic pathology, as determined by PSR staining for collagen, consists of highly cellular interstitial infiltrates with areas of diffuse fibrosis interspersed with fibrotic foci, adjacent to areas of unaffected tissue. The presence of contractile myofibroblasts within fibrotic foci can be visualized based on intense positive staining for alpha smooth muscle actin (*α*‐SMA) fibers (Fig. 1, day 17).

Representative images of lung tissue, taken at various time points postbleomycin challenge, are shown (Fig. 1). Tissue sections obtained from an early time point (day 3) displays signs of acute lung injury, with alveolar spaces filled with edematous fluid. By day 7, fibrosis is established, with fibrotic foci containing *α*‐SMA‐positive contractile myofibroblasts clearly visible within areas of increased cellularity and diffuse collagen fiber deposition. There is evidence of marked alveolar epithelial cell injury with loss of type I AEC and enlargement of the alveolar spaces to form cyst‐like structures (day 14). This corresponds to an increase in apoptosis within whole lung tissue, with a peak at day 10 post bleomycin, as detected by western blot analysis for expression of active caspase 3 relative to full‐length caspase 3 and GAPDH (data not shown). Areas of intense collagen fiber deposition can be seen (day 31: *arrow*), adjacent to sites of alveolar epithelial cell (AEC) injury. The alveolar septal thickening observed (day 31: *asterisk*) is associated with type II AEC hyperplasia (Fig. 1).

**Figure 2.**
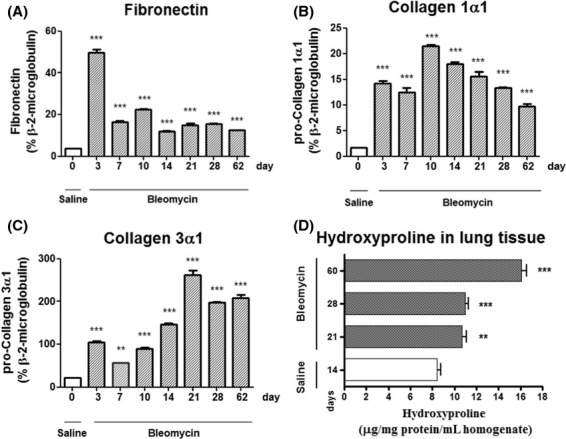
Bleomycin‐induced pulmonary fibrosis is associated with increased synthesis and deposition of extracellular matrix components within lung tissue. Gene transcript levels for the matrix components; (A) fibronectin, (B) collagen 1*α*1, (C) collagen 3*α*1, as determined by quantitative TaqMan PCR, relative to *β*2 microglobulin expression. (D) Levels of Hydroxyproline in lung tissue reflect enhanced collagen deposition and accumulation over time. Data are presented as mean ± SEM for groups of saline‐ or bleomycin‐challenged animals (*n *= 8) at various time points. Statistical significance (*P < 0.05, **P < 0.01, ***P < 0.001) determined using one‐way ANOVA.

Bleomycin‐induced fibrosis is thought to arise as a result of an acute lung injury response, associated with epithelial and endothelial cell apoptosis, with a loss in alveolar capillary barrier function, influx of a protein rich exudate, and deposition of a provisional matrix within the collapsed alveoli. Fibronectin is a principle component of this provisional matrix. Gene transcript analysis revealed significant (***P < 0.001) increases in fibronectin mRNA levels at various time points postbleomycin challenge, with a peak in expression on day 3, coinciding with the acute lung injury response (Fig. 2A).

Gene transcript analysis for collagen 1*α*1 and collagen 3*α*1 revealed significant (***P < 0.001) increases in mRNA levels in the lungs of bleomycin‐challenged rats, with active fibrosis and gene transcription on day 62 post challenge (Fig. 2B,C). The progressive nature of the fibrosis is demonstrated by significant (**P < 0.01) increases in levels of hydroxyproline; a principle amino acid of collagen, in lung tissue obtained from bleomycin‐challenged rodents over time. These data are suggestive of increased collagen synthesis and/or impaired collagen degradation in the lungs of bleomycin‐challenged rats (Fig. 2D).

### A prevailing profibrogenic microenvironment within the lungs of bleomycin‐challenged rats promotes fibrosis and extracellular matrix deposition

**Figure 3.**
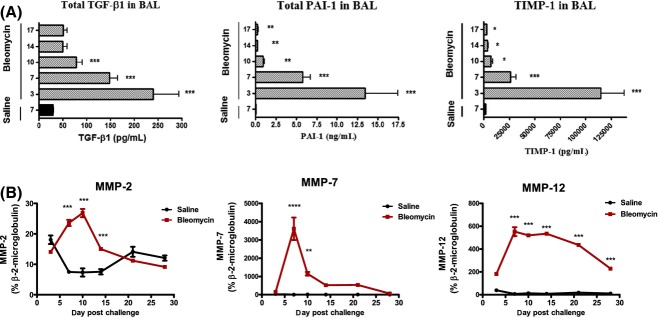
Levels of profibrotic mediators and metalloproteases are elevated in bronchoalveolar lavage (BAL) fluid and lung tissue following bleomycin challenge. (A) Levels of profibrotic mediators, TGF‐*β*1, PAI‐1, and TIMP‐1 in BAL fluid obtained at various time points postbleomycin or saline challenge, as determined by ELISA, following adjustment for protein levels. (B) Gene transcript levels for the metalloproteases, MMP‐2, MMP‐7, and MMP‐12 in lung tissue as determined by quantitative TaqMan PCR, relative to *β*2 microglobulin expression. Data are presented as mean ± SEM for each group (*n *= 8). Statistical significance (*P < 0.05, **P < 0.01, ***P < 0.001) as determined using one‐way ANOVA.

Here, we demonstrate significant (*P < 0.05 to ***P < 0.005) increases in mediators associated with fibrosis and vascular remodeling in bronchoalveolar lavage (BAL) fluid obtained from bleomycin‐challenged rats as compared to saline‐challenged controls. A peak in expression of protein levels for TGF‐*β*1 as well as the products of downstream target genes, plasminogen activator inhibitor (PAI)‐1 and tissue inhibitor of matrix metalloproteases (TIMP) ‐1, were observed on days 3–7, that is, preceding onset of established fibrosis (Fig. 3A). Elevated levels of matrix metalloproteases (MMPs) are associated with enhanced matrix turnover. Analysis of gene transcript levels in lung tissue revealed a significant (***P < 0.001), but transient, peak in mRNA levels for the proteases MMP‐2 (gelatinase A) and MMP‐7 (matrilysin). Levels of the predominantly macrophage‐derived metalloelastase, MMP‐12, remained significantly (***P < 0.001) elevated over time (Fig. 3B).

### Bleomycin‐challenged rats develop marked remodeling of the pulmonary vasculature and associated increases in right ventricular pressure

**Figure 4.**
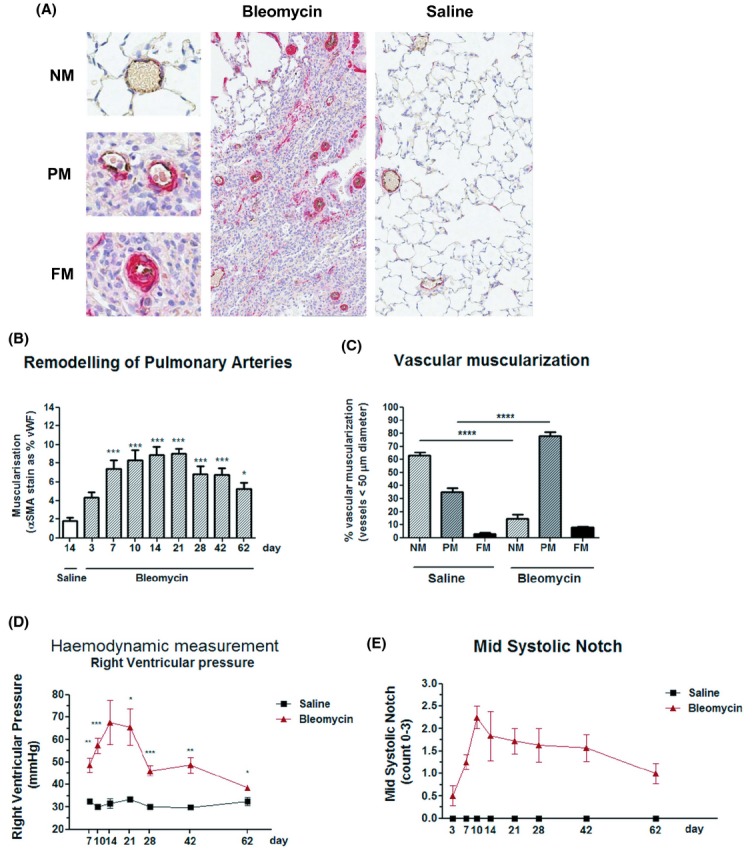
Bleomycin‐challenged rats display marked remodeling of the pulmonary vasculature within fibrotic regions of the lung, with associated increases in right ventricular pressure. (A) Immunohistochemical analysis of representative lung sections (day 21 post saline or bleomycin challenge) costained with antibodies specific for the endothelial marker von Willebrand factor (vWF: brown) or *α*‐smooth muscle actin (SMA: red) as a marker of vascular remodeling. Vessels categorized as nonmuscularized (NM), partially muscularized (PM) or fully muscularized (FM), with representative vessels shown. The extent of remodeling of small (< 50 *μ*m in diameter) pulmonary vessels within the lung was determined using either (B) image analysis software (image pro); and defined as percent of *α*‐SMA staining of vWF‐positive vessels, or (C) by morphometric analysis, with percent NM, PM or FM vessels analyzed in a blinded fashion for each section. The extent to which vascular remodeling has an impact on right ventricular pressure was determined (D) using direct hemodynamic measurements of right ventricular (RV) pressure, or (E) indirect measurements obtained by transthoracic echocardiography, with pulse wave doppler profiles of blood flow through the pulmonary artery valve used to determine mid‐systolic notching (scored 0–3). The data are presented as mean SEM for each group (*n *= 8). Statistical significance (*P < 0.05, **P < 0.01, ***P < 0.001) determined using one‐way ANOVA.

Bleomycin‐challenged rats display marked remodeling of the pulmonary vasculature within fibrotic regions of the lung, with associated increases in right ventricular pressure. The extent of remodeling of the microvasculature within the lung was determined by immunohistochemical analysis for the degree of *α*‐SMA staining by vascular smooth muscle cells (VSMC) surrounding vWF‐positive endothelial cells that form the walls of the vessel lumen (Fig. 4A). As in clinical PH and in contrast to pulmonary arterial hypertension (PAH), the formation of complex plexiform lesions is not observed. Using image analysis software, we were able to demonstrate a significant (***P < 0.001) increase in the percent of muscularized pulmonary arterioles (vessels ≤50 *μ*m in diameter) within the pulmonary interstitium, at various time points postbleomycin challenge, as compared to saline controls (Fig. 4B).

By manually scoring vessels throughout the entire section of lung tissue, we were able to establish that there were significant (****P < 0.0001) increases in the number of partially muscularized (PM) vessels in the lungs of bleomycin‐challenged rats, with a significant reduction in the number of nonmuscularized vessels (NM) and a trend toward an increase in the number of fully muscularized (FM) vessels (Fig. 4C). Many of these fully muscularized vessels display near total luminal occlusion (Fig. 4A).

Increases in vessels density are observed within areas of interstitial fibrosis, but not within the fibrotic foci themselves, where there is evidence for marked vessel ablation (Fig. 4A).

To determine the impact of vascular remodeling on right ventricular (RV) pressure, we performed right heart catheterization and direct hemodynamic measurements. Significant (0.05 > *P *< 0.001) increases in RV pressure were observed in bleomycin‐challenged rats as compared to saline‐challenged controls (Fig. 4D). The peak in RV pressure at earlier time points (days 10–28) may reflect the additive effect of low‐level inflammation on the underlying remodeling response. Noninvasive transthoracic (Doppler) echocardiography was performed in order to assess the extent of mid‐systolic notching as an estimate of blood flow through the pulmonary artery (tricuspid regurgitation). We observed a marked trend toward an increase in mid‐systolic notch (Fig. 4E) and increases in systolic RV pressure (data not shown). Despite the progressive nature of the fibrosis, we observe a gradual decline in RV pressure measurements, suggesting that as observed in high‐altitude‐induced PH in patients and in rodent models of hypoxia induced PH, the vascular remodeling response, seen here, is reversible and therefore amenable to therapeutic intervention.

### The pulmonary fibrosis and vascular remodeling induced on bleomycin challenge is associated with an increase in bone‐marrow‐derived macrophages, mast cells, and circulating tissue infiltrating fibrocytes

**Figure 5.**
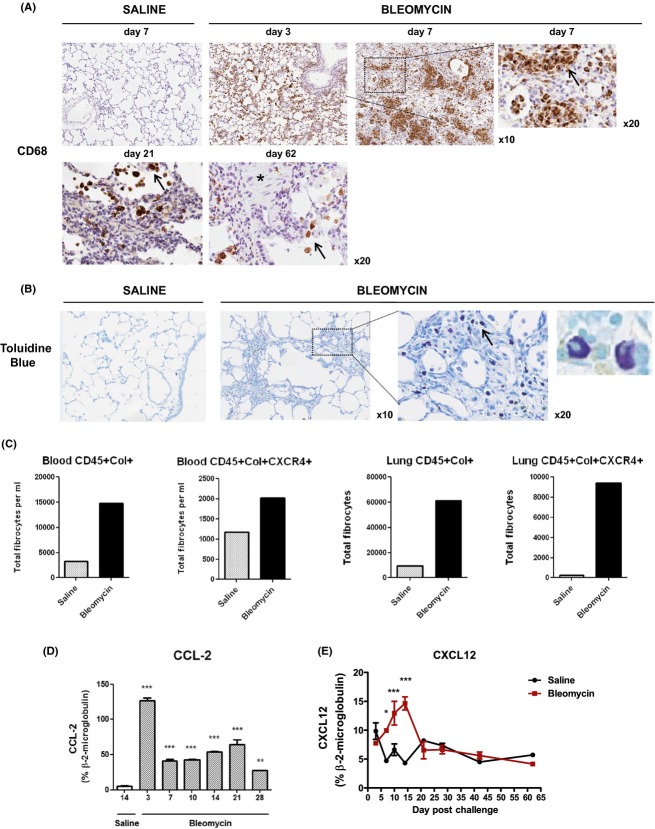
Recruitment of circulating hematopoietic cells to sites of pulmonary injury, fibrosis, and vascular remodeling. (A) Immunohistochemical analysis of lung sections from bleomycin‐challenged rats demonstrating infiltrating of CD68‐positive macrophages within early lesions at sites of lung injury and onset of fibrosis (days 3–7), with marked accumulation around vessels. CD68‐positive cells confined to alveoli at latter time points (day 21–62). (B) Histological analysis of lung sections stained with toluidine blue, demonstrating a marked infiltrate of activated degranulating mast cells within established fibrotic lesions (day 17 post bleomycin). Representative sections are shown (×10, ×20 magnification). (C) Flow cytometric analysis of pooled blood and lung tissue obtained from saline‐ or bleomycin‐challenged rats (day 14) demonstrating increases in circulating and lung tissue infiltrating fibrocytes as detected by costained for CD45, intracellular collagen I and CXCR4. Gene transcript analysis using TaqMan PCR demonstrate significant increases in levels of the chemokines (D) CCL‐2 and (E) CXCL12 in lung tissue at various time points following bleomycin challenge. Data are presented as mean SEM for each group (*n *= 8), with statistical significance (**P < 0.01, ***P < 0.001) determined using ANOVA.

Using immunohistochemical analysis, we demonstrate a marked perivascular infiltrate of CD68‐positive macrophages, localized to sites of injury and fibrosis within the lungs of bleomycin‐challenged rats (day 7). Increases in CD68‐positive cells were visible during the injury response (day 3), with greatly reduced numbers present at later time points, primarily within the alveoli and surrounding vessels. (Fig. 5A).

Increases in activated degranulating mast cells within established fibrotic lesions were observed, using histological staining of lung tissue with toluidine blue. Tissue sections shown here for day 17 post bleomycin (Fig. 5B).

Fibrocytes are circulating bone–marrow‐derived progenitor cells of mesenchymal origin that are thought to give rise to fibroblasts at sites of tissue injury and repair. In the absence of unique cell surface markers, fibrocytes are defined by flow cytometric analysis based on coexpression of the pan‐leukocyte cell surface marker CD45 and the intracellular mesenchymal marker procollagen I. Following initial sequential gating, subsets of fibrocytes were further defined based on the expression of the chemokine receptor CXCR4. Here, we demonstrate a marked increase in fibrocyte numbers in the systemic circulation and lung tissue of bleomycin‐challenged rats as compared to saline‐challenged controls (Fig. 5C). CXCR4 expression appears to be preferentially upregulated by CD45^+^ Collagen I^+^ fibrocytes within lung tissue, as compared to the systemic circulation, corresponding to localized increases in CXCL12 gene transcript expression levels (Fig. 5E) and suggesting a role in the recruitment of fibrocytes to sites of injury and repair. Data are presented for day 14 postbleomycin challenge, with fold increases in total fibrocyte numbers in bleomycin versus saline challenge shown [CD45^+^ Collagen‐I ^+^: circulation (4.5 fold), lung (6.5 fold) and CD45^+^ Collagen‐1^+^ CXCR4^+^: circulation (1.7 fold), lung (42.0 fold)] (Fig. 5C).

Circulating monocytic bone‐marrow‐derived precursor cells, including fibrocytes and monocytes, which may give rise to tissue‐resident fibroblasts and macrophages, respectively, are recruited into tissue along a chemokine gradient. Numerous studies have implicated a role for chemokine signaling pathways in disease pathophysiology. Using gene transcript analysis of lung tissue, we demonstrate significant increases in CCL‐2/MCP‐1 mRNA levels, with a peak in expression levels at day 3 post bleomycin (Fig. 5D). In contrast, mRNA levels of CXCL12/SDF‐1*α*, which is the ligand for CXCR4, were only transiently increased with significant (***P < 0.001) increases from days 7 to 14, returning to baseline by day 21 post bleomycin (Fig. 5E) and corresponding to the increase in fibrocyte numbers in lung tissue (Fig. 5C).

### Gene transcript analysis reveals significant increases in mechanistic pathway components associated with fibrosis and vascular remodeling

To assess whether the pathology observed in the bleomycin model reflects known mechanisms underlying clinical fibroproliferative disease and vascular remodeling, we investigated gene transcript levels for components of signaling pathways known to play a role in the pathogenesis of clinical IPF and PH.

**Figure 6.**
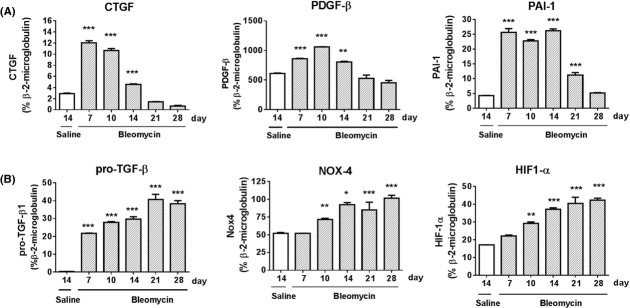
Gene transcript analysis of lung tissue demonstrating increased expression of profibrotic mediators associated with pathways linked to fibrosis and vascular remodeling in bleomycin‐challenged rats. Quantitative TaqMan PCR analysis of lung tissue obtained from saline‐ or bleomycin‐challenged rats, at various time points, demonstrates increased mRNA expression levels for (A) CTGF, (B) PDGF‐*α*, (C) PDGF‐*β*, (D) pro‐TGF‐*β*1, (E) PAI‐1, (F) HIF‐1*α*. Data are presented as mean ± SEM for each group (*n *= 8), with statistical significance (**P < 0.01, ***P < 0.001) as determined using one‐way ANOVA.

Here, we demonstrate significant (***P < 0.001) but transient (days 7–14 post bleomycin) upregulation in mRNA levels for the growth factors CTGF (Fig. 6A), PDGF‐*α* (data not shown; less than two fold maximal increase [day 14: Saline: Ct 25.95 ± 0.03, Bleomycin: Ct 34.73 ± 0.14)], and PDGF‐*β* (Fig. 6B), with the *β* isoform being the predominant form expressed in lung tissue. Gene transcripts for components of the TGF‐*β* signaling pathway, including pro‐TGF‐*β*1 (Fig. 6D) and the downstream target genes PAI‐1 (Fig. 6C), Nox‐4 (Fig. 6E), and HIF‐1*α* (Fig. 6F), were also significantly elevated in lung tissue post bleomycin, with sustained upregulation in expression levels over time.

**Figure 7.**
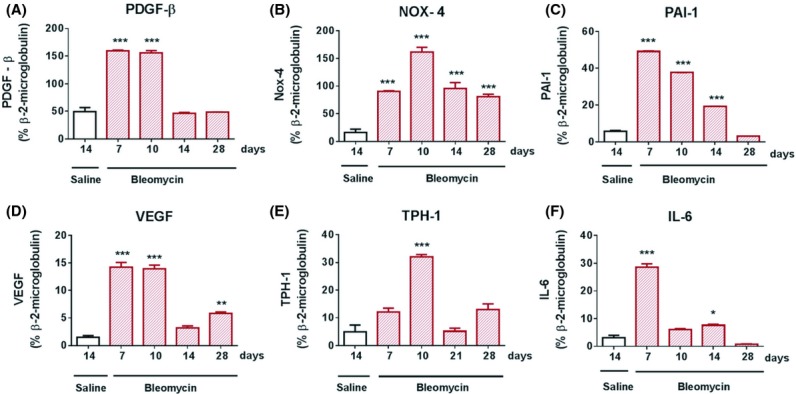
Gene transcript analysis of pulmonary arteries demonstrating increasing expression of mediators associated with vascular remodeling, in bleomycin‐challenged rats. Quantitative TaqMan PCR analysis of dissected pulmonary arteries obtained from saline‐ or bleomycin‐challenged rats, at various time points, demonstrates increased mRNA expression levels for (A) PDGF‐*β* (B) Nox‐4, (C) PAI‐1, (D) VEGF, TPH‐1, and IL‐6. Data are presented as mean ± SEM for each group (*n *= 8), with statistical significance (*P < 0.01, **P < 0.01, ***P < 0.001), as determined using one‐way ANOVA.

Within pooled microdissected pulmonary arteries, we observed significant (***P < 0.001) increases postbleomycin challenge, in mRNA expression levels, for the mediators associated with pulmonary hypertension in clinical disease and rodent disease models; PDGF‐*β* (Fig. 7A), Nox‐4 (Fig. 7B), PAI‐1 (Fig. 7C), VEGF (Fig. 7D), Tryptophan hydroxylase‐1 (TPH‐1) (Fig. 7E), and IL‐6 (Fig. 7F). Gene transcripts for Nox‐4, which is a component of the TGF‐*β*1 signaling pathway, remained significantly elevated within the pulmonary artery over time post bleomycin.

### Therapeutic treatment of bleomycin‐challenged rats with an ALK‐5 inhibitor compound targeting the TGF‐*β* signaling pathway causes a significant dose‐dependent reduction in the decline in lung function

**Figure 8.**
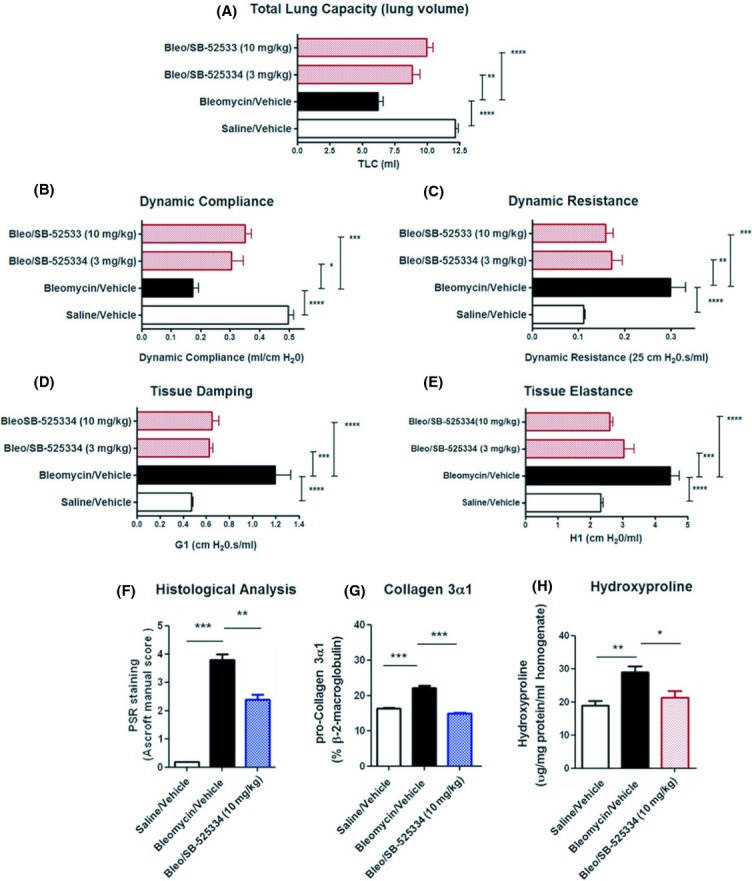
Application of the bleomycin model in evaluating the efficacy of therapeutic treatment with compounds targeting key components of signaling pathways involved in the pathogenesis of pulmonary fibrosis. (A) Lung function measurements for (A) total lung capacity (B) dynamic resistance, (C) dynamic compliance, (D) tissue damping as an index of in‐homogeneity in airflow in peripheral airways, and (E) tissue elastance in the peripheral airways were performed in order to investigate the impact of therapeutic treatment with the ALK‐5 small molecule inhibitor compound: SB‐525334 (3, 10 mg/kg/day) or vehicle alone, on an established fibrotic response in the bleomycin model. Animals were dosed with compound or vehicle, from day 7 onwards and after the appearance of pathological fibrotic lesions. The pulmonary pathology was assessed by morphometric analysis of Picro‐Sirius red (PSR)‐stained tissue sections using the Ashcroft scoring system (F), gene transcript levels for collagen 3*α*1 as determined by quantitative TaqMan PcR (G) and hydroxyproline content (H) in lung tissue. The data are presented as mean ± SEM for each group (*n *= 8), with statistical significance (*P < 0.05, **P < 0.01, ***P < 0.001), as determined using one‐way ANOVA.

Bleomycin‐challenged rats develop a significant (****P < 0.0001) decline in lung function with reductions in total lung capacity (Fig. 8A) and dynamic compliance (Fig. 8B) and increases in dynamic resistance (Fig. 8C) and in‐homogeneity in airflow within the lung parenchyma, including tissue damping (Fig. 8D) and tissue elastance (Fig. 8E). Here, we demonstrate that therapeutic treatment, after the onset of fibrosis, with the ALK‐5 (T*β*RI) inhibitor compound SB‐525334 (3 mg/kg/day, 10 mg/kg/day), resulted in significant dose‐dependent improvements in all parameters of lung function measured, as compared to vehicle‐treated controls. These improvements in lung function correlated with a significant reduction in pulmonary pathology as assessed by morphometric analysis using the clinical Ashcroft scoring system (**P < 0.001: Fig. 8F), gene transcript analysis for Collagen 3*α*1 (***P < 0.001: Fig. 8G) and measurement of hydroxyproline content (*P < 0.05: Fig. 8H), in bleomycin challenge rats treated therapeutically with SB‐525334 as compared to vehicle control. These data suggest a critical role for TGF‐*β*‐mediated responses in pulmonary fibrosis and associated decline in lung function.

### Therapeutic treatment of bleomycin‐challenged rats with either the ALK‐5 inhibitor SB‐525334 or Imatinib attenuated the increases in RV hypertrophy and RV pressure associated with pulmonary fibrosis

Bleomycin‐challenged rats develop significant increases in RV wall hypertrophy (*P < 0.05) and RV pressure in excess of >50 mmHg (***P < 0.001), as assessed by echocardiography and direct hemodynamic measurement, respectively. Baseline RV pressure readings in saline‐challenged animals were <25 mmHg.

**Figure 9.**
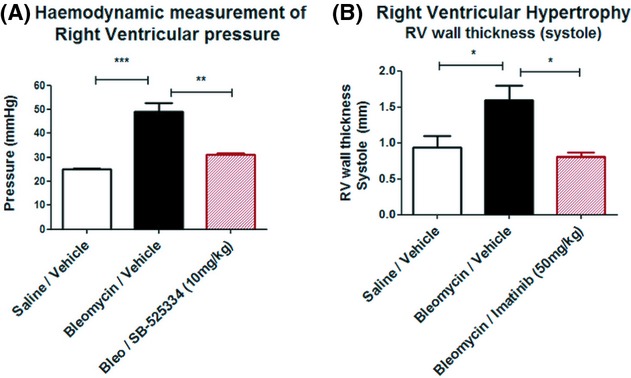
Application of the bleomycin models in evaluating the efficacy of therapeutic treatment with compounds targeting key components of signaling pathways involved in the pathogenesis of pulmonary hypertension associated with interstitial pulmonary fibrosis. (A) Hemodynamic measurements of right ventricular (RV) pressure by right heart catheterization and (B) transthoracic echocardiography measurements of right ventricular wall thickness (systole) were performed in order to investigate the impact of therapeutic treatment with either (A) the ALK‐5 inhibitor SB‐525334 (10 mg/kg/day) or (B) the PDGFR tyrosine kinase inhibitor imatinib (50 mg/kg/day). Animals were dosed with compound or vehicle from day 7 onwards, after the appearance of remodeled vasculature and onset of clinically significant increases in RV pressure. The data are presented as mean ± SEM for each group (*n *= 8), with statistical significance (*P < 0.05, **P < 0.01, ***P < 0.001) as determined using one‐way ANOVA.

Here, we demonstrate significant reductions in hemodynamic RV pressure measurements (**P < 0.01) in groups of bleomycin‐challenged rats treated therapeutically (day 7 onwards) with the small molecule ALK‐5 inhibitor compound SB‐525334 (10 mg/kg/day), as compared to vehicle‐treated controls (Fig. 9A). Similarly, we observe a significant reduction in increases in RV wall thickening (*P < 0.05) in groups of bleomycin‐challenged rats treated therapeutically with the tyrosine kinase inhibitor imatinib mesylate (50 mg/kg/day), as compared to the vehicle control group (Fig. 9B). These data suggest a critical role for TGF‐*β* and PDGFR signaling pathways in vascular remodeling and increases in RV pressure associated with interstitial pulmonary fibrosis.

## Discussion

Idiopathic pulmonary fibrosis (IPF) and PH remain diseases of high mortality and unmet medical need, despite recent advances in our understanding of mechanisms underlying disease pathogenesis. Animal models exhibiting principle pathophysiological features of IPF:UIP (American Thoracic Society, European Respiratory Society 2002) and PH [group 3.2 associated with chronic interstitial lung disease and alveolar hypoxia] (Proceedings of the 4th World Symposium 2009) could provide greater insight into common mechanistic pathways underlying disease pathogenesis and thereby facilitating the evaluation of novel targets and therapeutic approaches for intervention. Here, we describe a rodent model, which displays many of the principle pathophysiological features of clinical disease, including changes in physiological parameters used to clinically diagnose the extent of disease progression in patients; notably, a decline in lung function and increases in mPAP > 25 mmHg at rest (Galié et al. 2009b; Raghu et al. 2011).

Pulmonary hypertension (PH) is a pathophysiological parameter, encompassing a number of clinical conditions. No single animal model accurately reproduces all features of disease. The hypoxia and Sugen model aims to recapitulate processes driving the hyperproliferation of apoptosis‐resistant endothelial cells and plexiform arteriopathy, by VEGF receptor inhibition. The Monocrotaline model is characterized by vascular remodeling associated with a marked inflammatory infiltrate triggered by injury to the endothelium. The chronic hypoxia model exhibits a reversible pathology associated with remodeling of pulmonary arterioles due to SMC hyperplasia and hypertrophy. Here, we focused on PH secondary to chronic interstitial pulmonary fibrosis, in which hypoxic conditions, arising as a result of bleomycin induced alveolar epithelial cell injury and impaired gaseous exchange are thought to drive the pathological processes.

The fibrotic pathology is progressive, with evidence for sustained increases in collagen gene transcript levels that are suggestive of an active ongoing fibrotic response. This is in contrast to the routinely used C57BL/6 murine model of bleomycin‐induced fibrosis, in which the pathology is transient and which fails to recapitulate many of the principle features of clinical disease (Chung et al. 2003). The progressive nature of the fibrosis may reflect a prevailing profibrogenic environment within the lungs of bleomycin‐challenged rats, in which the accumulation of extracellular fibrillar collagen, at sites of injury, is due to increased collagen synthesis as well as impaired degradation of ECM components by proteolytic enzymes, such as plasmin and matrix metalloproteases (MMPs).

Plasminogen activator inhibitor (PAI)‐1 is responsible for inhibiting the enzymatic conversion of plasminogen to plasmin, resulting in reduced fibrinolytic activity and the accumulation of a fibrin‐rich provisional matrix at sites of alveolar injury, thus promoting the influx of inflammatory cells and fibroblasts. Levels of PAI‐1 are elevated in the lungs and BAL of patients with IPF (Stijn et al. 2013), as well as secondary PH. TGF‐*β*1, a principle mediator in the pathogenesis of IPF and PH, is thought to contribute toward disease pathology, both directly through transcriptional activation of ECM components such as collagen, as well as indirectly through upregulation of the transcription factor HIF‐1*α* which is responsible for the activation of downstream profibrotic target genes, such as PAI‐1 in fibroblasts (Cutroneo et al. 2007; Liu et al. 2010). Under conditions of hypoxia, PAI‐1 expression is upregulated in pulmonary macrophages, arterial smooth muscle cells (PASMC) and endothelial cells (Diebold et al. 2011), thereby providing a possible link between interstitial pulmonary fibrosis, alveolar hypoxia, and vascular remodeling.

Here, we observe increases in PAI‐1 mRNA and protein expression in lung tissue and BAL during the early time points following bleomycin challenge, which suggests that impaired fibrinolytic activity during acute or chronic inflammation leads to fibrosis and remodeling of the vasculature. Studies have shown that PAI‐1‐deficient mice are protected from bleomycin‐induced acute lung injury and fibrosis, whereas transgenic mice overexpressing PAI‐1 develop enhanced fibrosis (Eitzman et al. 1996). Notably, PAI‐1‐deficient mice are also protected from structural changes to the vasculature, including medial thickening of the vessel wall, in line with evidence for a critical role of PAI‐1 in promoting adhesion‐dependent growth and survival signals in VSMC and in protecting them from plasminogen activation–mediated apoptosis (Rossignal et al. 2006).

Matrix metalloproteases (MMPs), although ubiquitously expressed, are upregulated in chronic diseases, including IPF and PAH (Lepetit et al. 2005; Fusjishima et al. 2010). We observed increases in MMP‐2, MMP‐7, and MMP‐12 in the lungs of bleomycin‐challenged rats. MMPs are responsible for the degradation of ECM components including nonfibrillar collagen I, III, IV, and elastin. Collagen IV is a major component of the alveolar capillary basement membrane. Loss of basement membrane integrity, as a result of pulmonary injury, is thought to be decisive in predisposing toward a failure in epithelial regeneration and a loss of normal lung architecture and function that is characteristic of interstitial lung disease (Strieter 2008). In IPF patients, MMP‐2 expression was localized to myofibroblasts located in close proximity to areas of alveolar injury and basement membrane disruption, indicating a role in the influx of fibroblasts to sites of injury and alveolar collapse. In idiopathic PAH, elevated MMP‐2 expression by PASMC is thought to promote cellular migration and proliferation during vascular remodeling (Selman et al. 2000). Activated epithelial cells are responsible for increased MMP‐7 expression in IPF, with levels inversely correlating with a decline in FVC % and DL_CO_ % (Rosas et al. 2008). MMP‐7‐deficient mice are protected from bleomycin‐induced pulmonary fibrosis (Zuo et al. 2002). MMP‐12, released by macrophages infiltrating sites of injury and inflammation in the lung, is responsible for degradation of type IV collagen and elastin, suggesting a role in alveolar destruction and remodeling associated with the formation of honeycomb cyst–like structures (Sand et al. 2013). MMP‐12‐deficient mice are protected from alveolar epithelial cell apoptosis that proceeds alveolar septal thickening and fibrosis (Matute‐Bello et al. 2007).

Nevertheless, elevations in MMP gene expression do not correlate with activity, which is tightly regulated at transcriptional, translational, and post‐transcriptional levels. MMPs are secreted as inactive proenzymes, which are activated by proteases and ROS and which once active, are further regulated by TIMPs. Expression of TIMP‐1 is elevated in the lungs of IPF patients and is shown here to be elevated in BAL fluid postbleomycin challenge (Selman et al. 2000).

Pulmonary injury and inflammation are thought to play a key role in the pathogenesis of chronic interstitial lung diseases. In IPF, the relative importance of inflammation in clinical disease is disputed. Nevertheless, it is generally believed that inflammation may play a role in the early initiating subclinical phase of IPF (Strieter 2005), with low levels of inflammation persisting during established disease (Zuo et al. 2002). Here, we observe a pulmonary infiltrate consisting of CD68‐positive macrophages, localized to perivascular regions at sites of injury and fibrosis, as well as activated degranulating mast cells within areas of established interstitial fibrosis. We also observed increases in gene transcript levels for the chemokine CCL‐2 (MCP‐1), which is responsible for the recruitment of CCR2‐expressing monocytes and mast cells. Plasma levels of CCL‐2, released by the injured epithelium, are elevated in clinical IPF (Mercer et al. 2009). Studies in rodent disease models of bleomycin‐induced pulmonary fibrosis have demonstrated a critical role for alveolar macrophages in fibrotic responses (Gibbons et al. 2011). Activated macrophages produce a number of key pro‐inflammatory and profibrotic mediators including IL‐1*β*, TGF‐*β*, PDGF, and MMP‐12. In IPF, elevated numbers of mast cells within fibrotic lesions were shown to correlate with a decline in lung function. Immunohistochemical analysis confirmed expression of TGF‐*β*1 as well as the serine proteases, tryptase and chymase by MC_TC_ connective tissue mast cells, within fibrotic regions of the lung parenchyma and remodeled pulmonary arterioles (Andersson et al. 2011).

Inflammatory responses are also thought to play a role in the pathogenesis of PH, with evidence for perivascular inflammatory infiltrates consisting of mast cells and macrophages (Price et al. 2012; Savai et al. 2012). In rodent models of chronic hypoxia or monocrotaline (MCT)‐induced PH, the accumulation of inflammatory cells within the adventitia of pulmonary arteries is thought to play a key role in initiating intimal and medial remodeling of the vessel wall (Stenmark et al. 2013). Studies have shown that inhibition of mast cell recruitment and degranulation reduced the extent of vascular remodeling and associated increases in RV pressure (Dahal et al. 2011). Mast cell‐deficient Ws/Ws rats were largely protected from MCT‐induced PH (Hoffmann et al. 2011).

Numbers of circulating fibrocytes were also elevated in the lungs and peripheral blood of bleomycin‐challenged rats. Fibrocytes are circulating bone‐marrow‐derived hematopoietic progenitor cells that traffic to sites of injury, such as the lung, where they have the potential to differentiate into fibroblasts, thereby contributing toward fibrosis and remodeling. Numbers of circulating fibrocytes expressing the hematopoietic marker CD45 and the mesenchymal marker procollagen I as well as the chemokine receptor CXCR4 are increased in IPF (Mehrad et al. 2007; Andersson‐Sjoland et al. 2008) and PH (Yeager et al. 2012). In clinical PH, there is evidence for marked thickening of the pulmonary adventitia, due to fibroblast activation, proliferation, and differentiation into myofibroblasts. Studies in the chronic hypoxia calf model, in which the pathology most closely reflects human disease, identified a phenotypically distinct population of cells within the medial layer of remodeled pulmonary arteries, which express a fibrocyte‐like phenotype, including CD45, procollagen I, and the monocytic and progenitor cell markers CD14 and CD34/c‐kit. These cells are thought to contribute to vascular remodeling through the release of pro‐inflammatory mediators, mitogens, and growth factors; IL‐6, CCL‐2, CXCL12, PDGF (Frid et al. 2009). The role of CXCL12 in the recruitment of circulating fibrocytes to sites of chronic injury and repair was initially demonstrated in the bleomycin model of pulmonary fibrosis (Phillips et al. 2004). Levels of the ligand, CXCL12, are elevated in IPF patients and both primary iPAH and secondary PH (Mehrad et al. 2007; Andersson‐Sjoland et al. 2008; Yeager et al. 2012). Here, increases in circulating fibrocytes in peripheral blood and lung tissue correlated with gene transcript levels for CXCL12.

A number of growth factors have been implicated in the pathogenesis of fibrosis and vascular remodeling. In order to define which mediators are involved in the pathogenesis of vascular remodeling, we performed gene transcript analysis on pulmonary arteries dissected from bleomycin‐challenged rats. In addition to sustained increases in PDGF‐*β* and the TGF‐*β*1 pathway components, Nox‐4 and PAI‐1, we observed transient increases in VEGF, TPH‐1, and IL‐6, mediators associated with de‐regulated proliferation of activated microvascular endothelial cells and formation of plexiform lesions in primary PAH. VEGF is an endothelial cell–specific angiogenic mitogen, which has been shown to be upregulated in the lungs of patients with PAH, with expression localized to plexiform lesions (Hirose et al. 2000). IL‐6 is a pro‐inflammatory mediator, associated with increased mortality in iPAH. Transgenic mice overexpressing IL‐6 in the lung develop occlusive neointimal angio‐proliferative lesions, following exposure to hypoxia associated with elevated VEGF levels (Steiner et al. 2009). TPH‐1 is the rate‐limiting enzyme in the production of serotonin by pulmonary artery endothelial cells. In PAH, elevated levels of serotonin contribute toward pulmonary vascular constriction, increased proliferation of pulmonary artery smooth muscle cells, and vascular remodeling (MacLean and Dempsie 2010; Morecroft et al. 2012).

In the lung, the growth factors PDGF‐AA and ‐BB isoforms exhibit both chemoattractant and mitogenic properties for fibroblasts and SMC, as well as stimulating collagen synthesis by fibroblasts. The relative importance of the different isoforms, which exhibit overlapping biologic activities, remains to be defined. In IPF, PDGF‐BB appears to be the dominant isoform, with evidence for enhanced mitogenic activity and expression, predominantly by alveolar macrophages and to a lesser extent by type II AEC (Homma et al. 1995). In PH, early studies demonstrated significant increases in PDGF‐AA expression in iPAH lung tissue (Humbert et al. 1998). More recent studies using blood samples taken directly from the pulmonary artery during right heart catheterization revealed sustained increases in PDGF‐BB levels in patients, with primary and secondary PH (Selimovic et al. 2009). Here, we demonstrate increased levels of PDGF‐*α* and PDGF‐*β* mRNA in lung tissue obtained from bleomycin‐challenged rats, with predominant expression of the PDGF‐*β* isoform in lung and pulmonary arteries, during the early phase of the remodeling response. PDGF‐BB is known to be expressed within pulmonary arterioles by endothelial cells, smooth muscle cells, and perivascular inflammatory cells, including monocytes and macrophages. PDGF‐BB‐induced proliferation and migration of pulmonary arterial smooth muscle cells is inhibited by imatinib (Perros et al. 2008). Furthermore, therapeutic treatment with the tyrosine kinase inhibitor compound imatinib, which targets PDGFR signaling, as well as Abelson kinases (c‐ABL, BCR‐ABL), discoidin domain receptors (DDR) and c‐Kit signaling, significantly attenuated increases in RV pressure associated with vascular remodeling in bleomycin‐challenged rats. Similar results were observed in the rat MCT and murine chronic hypoxia models of PH (Schermuly et al. 2005). Although the efficacy of imatinib in reversing the remodeling response is assumed to be due to inhibition of PDGF receptor tyrosine kinase signaling, we cannot exclude the possibility that other mechanisms, including the inhibitory effects of imatinib on c‐kit tyrosine kinase signaling in circulating progenitor cells, such as fibrocytes and mast cells, may also have an impact.

In patients with advanced PAH, who fail to respond to conventional therapy, imatinib treatment leads to significant improvements in lung hemodynamics and RV function (Daniels et al. 2010). In contrast, a randomized, placebo‐controlled clinical trial in patients with mild to moderate IPF, failed to demonstrate any beneficial effect of imatinib on lung function or survival (Hoeper et al. 2013). This may reflect the lack of patient stratification or selection on the basis of presenting with secondary PH. Currently, there is no FDA approved treatment for IPF in general, or specifically for PH in patients with ILDs including IPF.

TGF‐*β*1 is a central mediator in the pathogenesis of both IPF and PH. Here, we observe significant increases in gene transcript levels for pro‐TGF‐*β*1 and the downstream target genes; Nox‐4, PAI‐1, and HIF‐1*α* in the lungs of bleomycin‐challenged rats. Sustained levels of TGF‐*β*1, Nox‐4, and HIF‐1*α* production are most likely due to the hypoxic microenvironment arising as a result of a loss in normal lung architecture leading to a decline in lung function and gaseous exchange. We previously demonstrated that the NADPH oxidase Nox‐4 is selectively upregulated in the lungs of patients with IPF and in bleomycin‐challenged rats. Nox‐4 is constitutively active, therefore, expression is directly associated with functional activity. Nox‐4‐derived reactive oxygen species act as an intracellular signaling molecule downstream of TGF‐*β* receptor ligation, responsible for activation of redox‐sensitive tyrosine kinases and transcription factors including HIF‐1*α* Inhibition of Nox‐4 activity attenuates TGF‐*β* induced responses associated with pulmonary fibrosis and vascular remodeling (Jarman et al. 2014). There is evidence for induction of Nox‐4 expression within the medial layer of remodeled pulmonary arteries, in patients with iPAH, reflecting a critical role for Nox‐4 in TGF‐*β*1‐mediated VSMC hyperplasia and hypertrophy (Mittal et al. 2007). Here, we demonstrate sustained increases in levels of Nox‐4 mRNA expression in the pulmonary arteries of bleomycin‐challenged rats, indicating a critical role of TGF‐*β* signaling in both interstitial fibrosis and vascular remodeling.

Studies have demonstrated that the ALK‐5 inhibitor compound SB‐525334, which targets TGF‐*β* signaling, prevented both the induction and progression of pulmonary fibrosis, when administered either prophylactically (Higashiyama et al. 2007) or therapeutically (Peng et al. 2013) to bleomycin‐challenged mice. Inhibition was assessed morphometrically as a reduction in the extent of staining for collagen I in lung tissue (Higashiyama et al. 2007; Peng et al. 2013). Similarly, targeting TGF‐*β*/ALK‐5 signaling in the model of MCT‐induced primary PH resulted in a small but significant improvement in the extent of vascular remodeling and in hemodynamic measurements of RV pressure (Zaiman et al. 2008).

Here, we demonstrate, using clinically translatable pathophysiological readouts of IPF and associated PH that therapeutic treatment of bleomycin‐challenged rats with the ALK‐5 inhibitor SB‐525334 attenuated the decline in lung function that is clinically associated with a loss in normal lung architecture and reversed to near normal levels, the increase in mPAP associated with vascular remodeling.

To our knowledge, this is the first study demonstrating the application of an animal model of interstitial lung disease, exhibiting pathophysiological features of both pulmonary fibrosis and associated pulmonary hypertension, as a means of evaluating the therapeutic potential of targeting common mechanistic signaling pathways associated with disease pathogenesis. For this purpose, the therapeutic efficacy of tool compounds were evaluated in this model using clinically translatable physiological readouts of lung function and hemodynamic measurement of mean PAP.

## Conflict of Interest

None declared.
